# Associations between COVID pandemic-related post-traumatic stress disorder symptoms and self-care behaviors, fear of hypoglycemia, and depressive symptoms among Chinese adults with type 2 diabetes in the post-pandemic era

**DOI:** 10.1186/s12888-025-07324-y

**Published:** 2025-09-30

**Authors:** Dianjiang Li, Enchun Pan, Ming Su, Zhongming Sun, Jinbo Wen, Keke Liu, Jingya Han, Xin Wang, Hong Fan, Sijun Liu

**Affiliations:** 1 Department of Social Medicine and Health Education, School of Public Health, Nanjing Medical University, Nanjing, China; 2 Department of Chronic Disease Prevention and Control, Huai’an City Center for Disease Control and Prevention, Huai’an, China; 3 Huai’an District Center for Disease Control and Prevention, Huai’an, China

**Keywords:** Type 2 diabetes, Post-traumatic stress disorder, Self-care behaviors, Hypoglycemia fear, Depressive symptoms, China

## Abstract

**Background:**

The COVID-19 pandemic significantly impacted individuals with type 2 diabetes (T2D), especially in China, where stringent public health measures disrupted healthcare access and heightened psychological stress. The long-term associations of pandemic-related post-traumatic stress disorder (PTSD) symptoms with self-care behaviors, fear of hypoglycemia (FoH), and depressive symptoms in T2D patients remain underexplored. This study examines these associations in Chinese adults with T2D in the post-pandemic period.

**Methods:**

We conducted a cross-sectional study with 242 adults with T2D at community health centers in Huai’an, China. Data on PTSD symptoms (Impact of Event Scale-Revised), self-care behaviors (Summary of Diabetes Self-Care Activities-6), FoH (Hypoglycemia Fear Survey II-Worry Scale), and depressive symptoms (Patient Health Questionnaire-9) were collected. Generalized linear models adjusted for demographic and clinical factors analyzed associations between PTSD symptoms and self-care, FoH, and depressive symptoms.

**Results:**

The prevalence of PTSD in the sample was 10.33% (*n* = 25). Higher PTSD scores were significantly associated with poorer dietary adherence (β = -0.0036, 95% CI: -0.0066 to -0.0005) and foot care (β = -0.0365, 95% CI: -0.0494 to -0.0235), as well as increased FoH (β = 0.0171, 95% CI: 0.0055 to 0.0287) and depressive symptoms (β = 0.0148, 95% CI: 0.0076 to 0.0220). No significant associations were found with physical activity, blood glucose testing, or medication adherence (all *P* > 0.05).

**Conclusions:**

COVID pandemic-related PTSD symptoms may be associated with certain worsened self-care behaviors, heightened FoH, and increased depressive symptoms among patients with T2D. These findings suggest that addressing mental health by integrating psychological support into chronic-care models could help improve diabetes outcomes, particularly for vulnerable groups facing future public health challenges.

**Supplementary Information:**

The online version contains supplementary material available at 10.1186/s12888-025-07324-y.

## Introduction

The COVID-19 pandemic, which began in late 2019, profoundly impacted global health, particularly individuals with chronic conditions like type 2 diabetes (T2D) [1]. In response, China implemented a rigorous Zero-COVID policy, involving mass testing, strict lockdowns, and border closures [2]. While these measures effectively controlled viral transmission over nearly three years, they also led to disruptions in healthcare access, routine disease management, and considerable mental health challenges [2]. Following the relaxation of this policy in December 2022, China experienced a significant surge in COVID-19 cases from December 2022 to February 2023 [3], which placed immense strain on healthcare systems and adversely affected patients with chronic conditions such as T2D [4].

Effective management of T2D requires consistent self-care behaviors, including adherence to a healthy diet, regular physical activity, blood glucose monitoring, and medication compliance [5]. However, the pandemic hindered many individuals’ ability to maintain these essential practices [6]. Research indicates that pandemic-induced stressors—such as fear of infection, social isolation, and uncertainty—created substantial barriers to effective diabetes management [7]. Additionally, individuals with T2D are often at increased risk for depression [8], which can further compromise their self-care practices [9]. Fear of hypoglycemia (FoH) also poses a significant concern, as it can deteriorate glycemic control and complicate overall disease management [10]. During the pandemic, the exacerbation of FoH may have been fueled by restricted healthcare access and ongoing uncertainty regarding health outcomes [11, 12].

The pandemic’s psychological impact led to the emergence of post-traumatic stress disorder (PTSD) in those exposed to its stressors [13]. PTSD, commonly associated with traumatic events, involves symptoms such as hyperarousal, intrusive thoughts, and avoidance, which can significantly impair daily functioning [14]. High rates of PTSD have been reported among those affected by the pandemic, with symptoms often rooted in fears of infection, prolonged isolation, and uncertainty about the future. For example, a study among Chinese university students during the pandemic found a 28% prevalence of posttraumatic stress symptoms [15], and another study reported a 19.9% prevalence of possible PTSD among frontline healthcare workers who survived COVID-19 six months after the outbreak [16]. These findings highlight the widespread psychological impact of the pandemic across different populations. Patients with chronic diseases, particularly those with T2D, were especially vulnerable to COVID pandemic-related PTSD due to their increased risk of severe outcomes and reduced access to routine care [17]. However, despite these high rates, there is a lack of research specifically examining the associations with patients with T2D, who may have unique vulnerabilities due to their health status and the challenges faced during the pandemic. Even though the acute phase of the pandemic has ended, its psychological effects persist, underscoring the need to investigate its lasting impact on chronic disease management.

Despite extensive research on the prevalence and impact of PTSD during the pandemic [18, 19], studies specifically examining its associations with self-care behaviors, FoH, and depressive symptoms in patients with T2D in the post-pandemic context remain limited. In China, where the prolonged Zero-COVID policy heightened the potential for lasting psychological effects, understanding these impacts among T2D patients is particularly pressing. This study seeks to examine the associations between COVID pandemic-related PTSD symptoms and key aspects of diabetes management, including self-care behaviors, FoH, and depressive symptoms, among a sample of T2D patients in China within the post-pandemic period. The findings are anticipated to shed light on the enduring psychological impact of the pandemic on diabetes management, offering valuable insights to better support this vulnerable population in future public health crises.

## Methods

### Study design and participants

This multicenter cross-sectional study was conducted at three community health service stations in Huai’an City, Jiangsu Province, China, under the National Essential Public Health Services Program (NEPHSP) focusing on the management of T2D. Eligible participants were all registered patients undergoing T2D management at these stations who met the World Health Organization (WHO) diagnostic criteria for T2D, were aged 35 years or older, and provided voluntary consent. Exclusion criteria included pregnancy, malignancy, a history of mental illness, or any serious medical condition that impaired communication or the ability to complete questionnaires. Data were collected between July and August 2023 through face-to-face interviews conducted by trained professionals. The professionals administered the survey using an electronic questionnaire on their mobile phones or tablet PCs. The questionnaire could only be submitted after all questions were answered and a logical consistency check was passed. Ultimately, 242 participants completed the survey. The study adhered to the Declaration of Helsinki and received approval from the Research Ethics Committee of Nanjing Medical University (NMU2019-716). All participants provided written informed consent. No financial or material incentives were provided to participants; participation was entirely voluntary.

The sample size was determined using G*Power software version 3.1.9.7 [20]. Assuming 11 predictors in a regression model, with a medium effect size of 0.15, a power of 0.80, and a significance level of 0.05, the minimum required sample size was calculated to be 127. The effect size of 0.15 was chosen as a medium effect size according to Cohen’s guidelines [21], providing a reasonable assumption for multiple regression in the absence of specific prior data. This requirement was exceeded, ensuring adequate statistical power for the study.

### PTSD symptoms

COVID pandemic-related PTSD symptoms were measured using the Impact of Event Scale-Revised (IES-R) [22]. The IES-R comprises 22 items across three subscales: intrusion, avoidance, and hyperarousal [23]. Participants rated their experiences related to stressful events caused by COVID-19 over the past seven days on a five-point Likert scale ranging from 0 (“not at all”) to 4 (“extremely”). Although the IES-R is not intended as a diagnostic instrument, it is one of the most extensively used screening measures for assessing the presence and severity of PTSD symptoms in both clinical and non-clinical settings [24]. Although originally designed to capture acute PTSD symptoms, the IES-R is widely used for both acute and chronic PTSD assessments, supporting its validity for longitudinal monitoring of symptom progression [25]. Total scores range from 0 to 88, with higher scores indicating more severe PTSD symptoms; a cut-off score of ≥ 33 suggests the presence of probable PTSD [23]. The IES-R has been translated into Chinese and validated in the literature [26], and it has been used in previous COVID-19 research in China [25]. In this study, the instrument demonstrated high internal consistency, with a Cronbach’s alpha of 0.909.

### Self-care behaviors

Diabetes self-care behaviors were assessed using the Summary of Diabetes Self-Care Activities-6 (SDSCA-6) [27]. This tool comprises seven items spanning six dimensions: healthy diet, physical activity, blood glucose testing (two items), foot care, and medication adherence. It measures the frequency of specific daily behaviors by assigning scores based on the number of days per week each behavior is performed. Each item is rated on a scale from 0 to 7, with higher scores indicating superior self-management. The psychometric properties of the SDSCA-6 have been validated for use in patients with diabetes [28].

### FoH

FoH in patients with T2D was evaluated using the Chinese adaptation of the Hypoglycemia Fear Survey II-Worry Scale (CHFSII-WS) by Mu et al. [29], which is derived from Cox et al.’s original worry subscale [30]. This instrument comprises 13 items distributed across two dimensions—worry and embarrassment—and utilizes a 5-point Likert scale ranging from 0 to 4. Scores range from 0 to 52, with higher scores indicating increased FoH. The CHFSII-WS was chosen for its validated reliability and applicability in T2D populations, capturing a diabetes-specific emotional response relevant to self-care behaviors [10]. In this study, the CHFSII-WS demonstrated high content validity, as evidenced by a Cronbach’s alpha coefficient of 0.975.

### Depressive symptoms

Depressive symptoms were assessed using the Patient Health Questionnaire-9 (PHQ-9), a nine-item self-report measure aligned with the Diagnostic and Statistical Manual of Mental Disorders, Fourth Edition (DSM-IV) criteria for major depressive disorder [31]. This instrument, validated for use in individuals with T2D [32], asks participants to rate the frequency of their symptoms over the past two weeks on a scale from 0 (“not at all”) to 3 (“nearly every day”). The total score is calculated by summing the item responses, yielding a range from 0 to 27, with higher scores indicating more severe depressive symptoms. In this study, the PHQ-9 demonstrated acceptable internal consistency, with a Cronbach’s α coefficient of 0.761.

### Other covariates

Data on sociodemographic and clinical characteristics were collected using a standardized questionnaire. Marital status was classified as either married or unmarried, with the latter including single, separated, divorced, or widowed individuals. Educational attainment was categorized into three levels: primary school or lower, junior school, and senior school or above. Residential areas were classified as urban or rural. The affordability of diabetes treatment was assessed by asking participants if their family could manage the medical expenses, with responses categorized as completely affordable, largely affordable, or hardly affordable.

COVID-19 history was determined through self-reported COVID-19 infection status over the past three years (yes, no, or unknown). Diabetes duration was calculated from the time of diagnosis to the age at examination. Glucose-lowering medications were grouped into four categories: only oral glucose-lowering drugs (OGLDs), only insulin, both OGLDs and insulin, and none as recommended by a physician. Hypoglycemia history (yes, no) was assessed based on self-reported incidents within the previous six months. Participants also listed any diabetes-related comorbidities, including nephropathy, retinopathy, neuropathy, coronary artery disease, stroke, peripheral arterial disease, and diabetic foot, with all conditions verified through official medical documentation.

These covariates were included to account for potential confounding, as previous studies have shown that factors such as gender, socioeconomic status (proxied by treatment affordability), and history of hypoglycemia can influence both PTSD symptoms and diabetes management outcomes [33, 34].

### Statistical analysis

Continuous variables with skewed distributions were reported as medians with interquartile ranges (IQRs), while categorical variables were described as frequencies with percentages. Participant characteristics were compared between the PTSD and non-PTSD groups using the Wilcoxon rank-sum test and Fisher’s exact test, as appropriate. No adjustments for multiple testing were made, as these comparisons were descriptive.

Generalized linear models were used to assess the associations between PTSD symptoms and outcomes, including self-care behaviors, FoH, and depressive symptoms. Given the skewed distributions of the continuous outcome variables (see Supplementary Table 1 for descriptive statistics and normality tests), generalized linear models were employed to accommodate non-normal data, aligning with methodological recommendations for health research [35, 36]. Three models were constructed: Model 1 was unadjusted; Model 2 was adjusted for age (continuous), gender, marital status, educational attainment, residential area, and diabetes treatment affordability; and Model 3 was further adjusted for diabetes duration (continuous), type of glucose-lowering medication, history of hypoglycemia, history of COVID-19, and the number of diabetes-related comorbidities (continuous). IES-R scores were analyzed as a continuous exposure (per-point increase) in all regression models, with the dichotomous PTSD status (IES-*R* ≥ 33) used only for descriptive subgroup comparisons.

Statistical significance was defined as a two-sided p-value of less than 0.05. All analyses were performed using SAS version 9.4 (SAS Institute Inc., Cary, NC, USA) on a Windows platform.

## Results

### Characteristics of participants

Table 1 presents the baseline characteristics of the 242 participants. The median age was 65.75 years (IQR: 57.00–70.67), with 64.46% aged 60 years or older. The median duration of diabetes was 8 years (IQR: 5–13). The majority were female (58.26%), married (86.78%), and lived in rural areas (75.62%). Over half reported complete affordability of diabetes treatment (51.24%), were prescribed only OGLDs (80.17%), and had a history of COVID-19 infection (61.98%). Additionally, 44.21% had an education level of primary school or lower, 37.6% had a history of hypoglycemia, and 44.63% had diabetes-related comorbidities.

**Table 1:** Baseline characteristics of the study participants

Variable	Overall	No probable PTSD (IES-*R* < 33)	Probable PTSD (IES-*R* ≥ 33)	*P* value
No. of participants	242	217	25	
Age, median (IQR), years	65.75(57.00,70.67)	65.67(56.75,70.83)	65.83(58.75,68.83)	0.900
35–59	86(35.54)	77(35.48)	9(36)	1.000
≥ 60	156(64.46)	140(64.52)	16(64)	
Gender				
Male	101(41.74)	96(44.24)	5(20)	0.030
Female	141(58.26)	121(55.76)	20(80)	
Education attainment				
Primary school and below	107(44.21)	94(43.32)	13(52)	0.474
Junior school	63(26.03)	59(27.19)	4(16)	
Senior school and above	72(29.75)	64(29.49)	8(32)	
Marital status				
Married	210(86.78)	187(86.18)	23(92)	0.546
Unmarried	32(13.22)	30(13.82)	2(8)	
Residential area				
Urban	59(24.38)	53(24.42)	6(24)	1.000
Rural	183(75.62)	164(75.58)	19(76)	
Diabetes treatment affordability				
Completely affordable	124(51.24)	118(54.38)	6(24)	0.010
Largely affordable	95(39.26)	80(36.87)	15(60)	
Hardly affordable	23(9.50)	19(8.76)	4(16)	
Diabetes duration, median (IQR), years	8(5,13)	8(5,13)	10(4,14)	0.498
Glucose-lowering medication types				
OGLDs only	194(80.17)	173(79.72)	21(84)	0.579
Insulin only	21(8.68)	20(9.22)	1(4)	
OGLDs plus insulin	23(9.5)	21(9.68)	2(8)	
None as advised by a physician	4(1.65)	3(1.38)	1(4)	
History of hypoglycemia				
Yes	91(37.6)	79(36.41)	12(48)	0.280
No	151(62.4)	138(63.59)	13(52)	
No. of diabetes-related comorbidities				
0	134(55.37)	122(56.22)	12(48)	0.670
1	83(34.3)	73(33.64)	10(40)	
2	20(8.26)	17(7.83)	3(12)	
3	5(2.07)	5(2.30)	0(0)	
History of COVID-19				
Yes	150(61.98)	133(61.29)	17(68)	0.714
No	80(33.06)	72(33.18)	8(32)	
Unknown	12(4.96)	12(5.53)	0(0)	
Self-care behaviors				
Healthy diet, median (IQR)	7(6,7)	7(6,7)	6(4,7)	0.133
Physical activity, median (IQR)	7(3,7)	7(3,7)	7(5,7)	0.786
Blood glucose testing, median (IQR)	0(0,1)	0(0,1)	0.5(0,1)	0.090
Foot care, median (IQR)	0(0,1)	0(0,1)	0(0,0)	0.317
Medication adherence^a^, median (IQR)	7(7,7)	7(7,7)	7(7,7)	0.803
FoH (CHFSII-WS), median (IQR)	0(0,9)	0(0,7)	10(1,13)	0.001
Depressive symptoms (PHQ-9), median (IQR)	3(1,5)	2(1,5)	8(3,10)	< 0.001

*PTSD* Post-traumatic stress disorder, *IES-R* Impact of Event Scale-Revised, *IQR* Interquartile range, *OGLDs* Oral glucose-lowering drugs, *FoH* Fear of hypoglycemia, *CHFSII-WS* Chinese version of Hypoglycemia Fear Survey II-Worry Scale, *PHQ-9* Patient health questionnaire-9

^a^Excluding participants who, as advised by their physicians, were not taking glucose-lowering medications (*n* = 4)

The median scores for healthy diet, physical activity, blood glucose testing, foot care, and medication adherence were 7 (IQR: 6–7), 7 (IQR: 3–7), 0 (IQR: 0–1), 0 (IQR: 0–1), and 7 (IQR: 7–7), respectively. The median scores for the CHFSⅡ-WS and PHQ-9 were 0 (IQR: 0–9) and 3 (IQR: 1–5), respectively.

### Prevalence of PTSD

The overall prevalence of PTSD (IES-R total score ≥ 33) in the sample was 10.33% (*n* = 25). As shown in Table 1, participants in the PTSD group were more likely to be female (*P* = 0.03) and had significantly higher median CHFSⅡ-WS (*P* = 0.001) and PHQ-9 scores (*P* < 0.001) compared to the non-PTSD group. Additionally, participants without PTSD were more likely to report complete affordability of diabetes treatment (*P* = 0.01).

### Association of PTSD symptoms with self-care behaviors

In generalized linear models, after adjusting for age, gender, marital status, educational attainment, residential area, diabetes treatment affordability, diabetes duration, type of glucose-lowering medication, history of hypoglycemia, history of COVID-19, and the number of diabetes-related comorbidities, the IES-R score was significantly negatively associated with a healthy diet (β = −0.0036, 95% CI: −0.0066 to −0.0005) and foot care (β = −0.0365, 95% CI: −0.0494 to −0.0235) (Table 2). However, no significant associations were found between the IES-R score and physical activity, blood glucose testing, or medication adherence (all *P* > 0.05; Table 2).

**Table 2:** Association of PTSD symptoms with self-care behaviors by using generalized linear models (*n* = 242)

	Healthy diet		Physical activity		Blood glucose testing		Foot care		Medication adherence^a^	
	*β (95% CI)*	*P* value	*β (95% CI)*	*P* value	*β (95% CI)*	*P* value	*β (95% CI)*	*P* value	*β (95% CI)*	*P* value
Model 1	−0.0032(−0.0059,−0.0004)	0.024	0.0012(−0.002,0.0044)	0.477	0.0059(−0.0056,0.0174)	0.317	−0.0283(−0.0397,−0.0168)	< 0.001	0.0003(−0.0009,0.0016)	0.588
Model 2	−0.0037(−0.0066,−0.0007)	0.017	0.0008(−0.0025,0.0042)	0.626	0.0025(−0.0094,0.0143)	0.683	−0.0272(−0.0401,−0.0143)	< 0.001	0.0003(−0.001,0.0017)	0.620
Model 3	−0.0036(−0.0066,−0.0005)	0.021	0.0005(−0.0029,0.0039)	0.782	0.002(−0.0104,0.0144)	0.754	−0.0365(−0.0494,−0.0235)	< 0.001	0.0004(−0.001,0.0018)	0.590

Model 1: unadjusted

Model 2: adjusted for age (continuous), gender, marital status, educational attainment, residential area, and diabetes treatment affordability

Model 3: further adjusted for diabetes duration(continuous), glucose-lowering medication types, history of hypoglycemia, history of COVID-19, and the number of diabetes-related comorbidities (continuous)

*PTSD* Post-traumatic stress disorder, *CI* Confidence interval

^a^Excluding participants who, as advised by their physicians, were not taking glucose-lowering medications (*n* = 4)

### Association of PTSD symptoms with FoH and depressive symptoms

In generalized linear models, when controlling for age, gender, marital status, educational attainment, residential area, diabetes treatment affordability, diabetes duration, glucose-lowering medication types, history of hypoglycemia, history of COVID-19, and the number of diabetes-related comorbidities, the IES-R score was significantly positively associated with FoH (β = 0.0171, 95% CI: 0.0055 to 0.0287) and depressive symptoms (β = 0.0148, 95% CI: 0.0076 to 0.0220) (Table 3).

**Table 3:** Association of PTSD symptoms with FoH, and depressive symptoms by using generalized linear models (*n* = 242)

	FoH (CHFSII-WS)		Depressive symptoms (PHQ-9)	
	*β (95% CI)*	*P* value	*β (95% CI)*	*P* value
Model 1	0.0132(0.0025,0.0239)	0.016	0.0172(0.0100,0.0245)	< 0.0001
Model 2	0.0161(0.0041,0.0280)	0.009	0.0162(0.0090,0.0233)	< 0.0001
Model 3	0.0171(0.0055,0.0287)	0.004	0.0148(0.0076,0.0220)	< 0.0001

Model 1: unadjusted

Model 2: adjusted for age (continuous), gender, marital status, educational attainment, residential area, and diabetes treatment affordability

Model 3: further adjusted for diabetes duration(continuous), glucose-lowering medication types, history of hypoglycemia, history of COVID-19, and the number of diabetes-related comorbidities (continuous)

*PTSD* Post-traumatic stress disorder, *FoH* Fear of hypoglycemia, *CHFSII-WS* Chinese version of Hypoglycemia Fear Survey II-Worry Scale, *PHQ-9* Patient health questionnaire-9, *CI* Confidence interval

## Discussion

To our knowledge, this study is the first to explore the associations between COVID pandemic-related PTSD symptoms and self-care behaviors, FoH, and depressive symptoms among a sample of T2D patients in Huai’an, China, in the post-pandemic era. By focusing on this vulnerable population, who faced disruptions in healthcare services and heightened psychological stressors during the pandemic, our research provides preliminary evidence of the long-term psychological impact of the pandemic on diabetes management.

The prevalence of PTSD in our sample (10.33%) is noteworthy, particularly considering that data collection occurred six months after the peak of the COVID-19 pandemic in China. During the pandemic, many studies reported a significant increase in PTSD rates, especially among high-risk groups. A meta-analysis of 14 cross-sectional studies (including four from China), conducted between March 11, 2020, and October 11, 2021, found a pooled PTSD prevalence of 30% among high-risk populations, such as pregnant women, individuals with chronic illnesses, and those undergoing hemodialysis [18]. Other meta-analyses reported varying PTSD rates among different groups during the pandemic: COVID-19 patients (28.34%) [37], college students (25%) [38], and the general population (17.34%) [39]. However, data on PTSD prevalence in the post-pandemic period are Limited. A study conducted in April 2023, two months after the peak in China, surveyed 2,513 nurses and found a PTSD prevalence of 41% [40]. In our study, the PTSD prevalence among individuals with T2D was lower than rates reported during the pandemic, particularly in high-risk groups, but still 2.5 times higher than the prevalence of 4.0% observed in the general population under normal conditions in 2017 [41]. This finding highlights a persistent psychological impact that has not fully subsided, even after the relaxation of stringent public health measures.

In this study, we found that higher levels of COVID pandemic-related PTSD symptoms were significantly associated with lower adherence to a healthy diet and foot care practices. These findings align with a meta-analysis of 29 studies, which showed that individuals with PTSD often exhibit disordered eating behaviors and poor diet quality [42]. This relationship may be attributed to using food as a coping mechanism for managing negative emotions, which can lead to unhealthy dietary habits and increase the burden of diabetes and related conditions [43]. Additionally, our study supports existing evidence that PTSD symptoms are linked to inadequate foot care practices. Previous research suggests that individuals who receive proper foot care guidance are more likely to maintain regular foot care routines [44]. PTSD symptoms such as hyperarousal and avoidance may contribute to neglecting preventive foot care unless explicitly guided by healthcare professionals. These findings underscore the potential need for healthcare providers to consistently assess and educate patients with PTSD symptoms on foot care to possibly improve diabetes management outcomes.

In contrast, our study did not find a significant association between COVID pandemic-related PTSD symptoms and physical activity, aligning with previous mixed findings. A review of 12 studies reported that about half found no significant relationship between PTSD and physical activity [42], while others indicated that trauma-exposed individuals with lower PTSD severity were more likely to engage in vigorous exercise [45]. One possible explanation for our findings is the high proportion of older adults in our sample (64.46% aged 60 or older). In these participants, factors such as age and age-related ailments may have had a more significant influence on exercise engagement than trauma or psychiatric variables [43]. Moreover, although a previous meta-analysis linked PTSD symptoms to non-adherence to chronic medication regimens [46], we did not observe this association. This discrepancy could be due to differences in trauma contexts; our study focused on PTSD related to COVID-19, while prior research examined PTSD resulting from acute medical events such as stroke or cancer [46]. Similarly, while a previous study reported a positive association between PTSD symptoms and self-monitoring of blood glucose [47], our results found no significant link. This divergence may result from variations in sample characteristics or individual differences in how PTSD affects vigilance in illness management, with some individuals exhibiting increased adherence and others showing neglect in diabetes self-care [47]. Additionally, the low overall frequency of blood glucose testing in our sample, consistent with prior findings in China where patients often rely on periodic clinic tests rather than daily home monitoring [48], may have limited our ability to detect an association.

The present study also reveals significant associations between PTSD symptoms, FoH, and depressive symptoms in individuals with T2D following the COVID-19 pandemic. PTSD-related hypervigilance and fear responses likely heighten concerns about hypoglycemia, particularly in diabetic patients [49], thereby complicating diabetes management. This finding aligns with previous research showing that individuals with high neuroticism are more susceptible to fear-related disorders [49], which can amplify fears and increase psychological distress [50]. Additionally, the relationship between PTSD and depressive symptoms reflects their well-documented comorbidity, often linked to shared dysregulation of the hypothalamic-pituitary-adrenal (HPA) axis, which increases stress sensitivity [51, 52]. Studies have shown a direct association between PTSD and depression [53], highlighting their reciprocal nature [54] and the persistence of each disorder in exacerbating the other [55]. This interconnectedness is fueled by shared symptoms such as sleep disturbances, anhedonia, and irritability, which perpetuate a cycle of chronic physiological dysregulation. The synaptic dysconnectivity model provides insight into this persistence by suggesting that chronic stress pathology results from trauma-induced changes in the prefrontal cortex and hippocampus, similar to the stress-related synaptic changes observed in depression [53]. Consequently, the shared pathophysiology may create a feedback loop in which depressive symptoms compound PTSD, intensifying psychological strain. The COVID-19 pandemic likely exacerbated these relationships, as pandemic-related trauma and isolation worsened PTSD and depressive symptoms, especially in vulnerable populations like diabetic patients [56]. Interestingly, among participants with probable PTSD, 17 had a history of COVID-19 infection, while 8 did not. This suggests that PTSD symptoms may not solely arise from direct infection but could also be influenced by broader pandemic-related stressors, such as disrupted healthcare access, social isolation, or general anxiety about health. Future research with larger samples should explore how direct infection versus indirect pandemic stressors contribute to PTSD in chronic disease populations. These findings emphasize the need for integrated healthcare approaches that address both mental and physical health, recognizing the lasting impact of pandemic-related PTSD and depression on diabetes management and overall well-being.

Despite these contributions, this study has limitations. First, the cross-sectional design prevents the establishment of causal relationships between PTSD symptoms and the observed outcomes. Longitudinal studies are essential to better understand how PTSD symptoms evolve and influence diabetes self-care over time. Second, as our sample was drawn from only three community health service stations in Huai’an City, the findings may not generalize to other regions or populations with different sociodemographic characteristics. The predominance of rural participants (75.62%) and lower educational attainment could influence self-care and psychological outcomes, limiting applicability to urban or more educated groups. Future studies should recruit more diverse samples across urban and rural settings to enhance generalizability. Third, we relied on self-report measures (IES-R, SDSCA-6, CHFSII-WS, PHQ-9), introducing potential recall and response biases that could affect the accuracy of the data. Fourth, we did not account for all possible confounders, such as social support or other mental health conditions, which might influence the associations observed. Finally, the use of the IES-R, primarily validated for acute trauma, may be less optimal for chronic PTSD assessment, though it remains suitable for long-term monitoring [25]. Future research could incorporate diagnostic tools for chronic PTSD and broader emotional measures like diabetes distress.

## Conclusion

This study reveals a notable prevalence of PTSD symptoms, potentially associated with challenges in diabetes self-care behaviors, heightened FoH, and increased depressive symptoms among a sample of T2D patients in China within the post-pandemic period. Specifically, individuals exhibiting COVID pandemic-related PTSD symptoms demonstrated lower adherence to healthy dietary practices and foot care, indicating that psychological distress can undermine essential self-care routines in diabetes management. Additionally, the positive associations between PTSD symptoms, FoH, and depressive symptoms underscore the interconnectedness of mental and physical health in managing chronic diseases, particularly in a post-pandemic context where individuals face long-term impacts of trauma. These results suggest the need for integrated care strategies that address both mental and physical health, especially within vulnerable populations impacted by COVID-19. Interventions targeting PTSD and depression may help alleviate the psychological burdens that could impede effective diabetes management, potentially enhancing the quality of life and health outcomes for this at-risk group, while also preparing for future public health crises.

## Supplementary Information

Supplementary Material 1.

## Data Availability

The datasets generated and/or analysed during the current study are available from the corresponding author on reasonable request.

## Abbreviations

CHFSII-WS: Chinese adaptation of the Hypoglycemia Fear Survey II-Worry Scale
CI: Confidence interval
FoH: Fear of hypoglycemia
IES-R: Impact of Event Scale-Revised
IQR: Interquartile range
NEPHSP: National Essential Public Health Services Program
OGLDs: Oral glucose-lowering drugs
PHQ-9: Patient Health Questionnaire-9
PTSD: Post-traumatic stress disorder
SD: Standard deviations
SDSCA-6: Summary of Diabetes Self-Care Activities-6
T2D: Type 2 diabetes
