# Supplementary material for: Associations between COVID pandemic-related post-traumatic stress disorder symptoms and self-care behaviors, fear of hypoglycemia, and depressive symptoms among Chinese adults with type 2 diabetes in the post-pandemic era
Source: BMC Psychiatry. 2025 Sep 30;25:892. doi: 10.1186/s12888-025-07324-y (PMC12487103; doi:10.1186/s12888-025-07324-y)
Supplement: Supplementary file 1 — Supplementary Material 1. [file 12888_2025_7324_MOESM1_ESM.docx]

**Associations between COVID pandemic-related post-traumatic stress disorder symptoms and self-care behaviors, fear of hypoglycemia, and depressive symptoms among Chinese adults with type 2 diabetes in the post-pandemic era**

**Supplementary Table 1 Descriptive Statistics of Variables (n=242)**

| **Variable** | **Minimum** | **Maximum** | **Mean** | **SD** | **Skewness** | **Kurtosis** | **P-Value (Shapiro-Wilk)** | **P-Value (Kolmogorov-Smirnov)** |
| --- | --- | --- | --- | --- | --- | --- | --- | --- |
| Healthy diet | 0 | 7 | 5.71 | 2.26 | -1.71 | 1.49 | <0.0001 | <0.01 |
| Physical activity | 0 | 7 | 4.97 | 2.91 | -0.93 | -0.96 | <0.0001 | <0.01 |
| Blood glucose testing | 0 | 7 | 0.77 | 1.49 | 2.54 | 6.62 | <0.0001 | <0.01 |
| Foot care | 0 | 7 | 0.96 | 2.04 | 2.15 | 3.37 | <0.0001 | <0.01 |
| Medication adherence | 0 | 7 | 5.96 | 2.38 | -2.02 | 2.27 | <0.0001 | <0.01 |
| FoH (CHFSⅡ-WS) | 0 | 51 | 5.15 | 8.35 | 2.17 | 5.52 | <0.0001 | <0.01 |
| Depressive symptoms (PHQ-9) | 0 | 21 | 3.55 | 3.63 | 1.45 | 2.53 | <0.0001 | <0.01 |
| PTSD symptoms (IES-R) | 0 | 58 | 14.45 | 13.58 | 0.86 | -0.08 | <0.0001 | <0.01 |

Note: SD, standard deviation; FoH, fear of hypoglycemia; CHFSⅡ-WS, Chinese version of Hypoglycemia Fear Survey Ⅱ-Worry Scale; PHQ-9, Patient health questionnaire-9.
